# Diagnostic model using LI-RADS v2018 for predicting early recurrence of microvascular invasion-negative solitary hepatocellular carcinoma

**DOI:** 10.1186/s40644-025-00865-1

**Published:** 2025-03-31

**Authors:** Yingying Liang, Xiaorui Han, Tingwen Zhou, Chuyin Xiao, Changzheng Shi, Xinhua Wei, Hongzhen Wu

**Affiliations:** 1 Department of Radiology, Guangzhou First People’s Hospital, Guangzhou Medical University, School of Medicine, South China University of Technology, 1Panfu Road, Guangzhou, Guangdong Province 510180 China; 2 Department of Radiology, The First Affiliated Hospital of Jinan University, Huangpudadaoxi, Guangzhou, Guangdong Province 510630 China

**Keywords:** Hepatocellular carcinoma, Magnetic resonance imaging, Model, Recurrence

## Abstract

**Objectives:**

To develop a diagnostic model for predicting the early recurrence of microvascular invasion (MVI)-negative hepatocellular carcinoma (HCC) after surgical resection, using the Liver Imaging Reporting and Data System (LI-RADS) version 2018.

**Methods:**

This retrospective study included 73 patients with MVI-negative HCC who underwent Gadoxetic acid-enhanced MRI (EOB-MRI) scanning before surgical resection. The clinical factors and LI-RADS v2018 MRI features associated with early recurrence were determined using univariable and multivariable analyses. A diagnostic model predicting early recurrence after surgical resection was developed, and its predictive ability was evaluated via a receiver operating characteristic curve. Then, the recurrence-free survival (RFS) rates were analyzed by Kaplan–Meier method.

**Results:**

In total, 26 (35.6%) patients were diagnosed with early recurrence according to the follow-up results. Infiltrative appearance and targetoid hepatobiliary phase (HBP) appearance were independent predictors associated with early recurrence (*p* < 0.05). For the established diagnostic model that incorporated these two significant predictors, the AUC value was 0.76 (95% CI: 0.64–0.85) for predicting early recurrence after resection, which was higher than the infiltrative appearance (AUC: 0.67, 95% CI: 0.55–0.78, *p* = 0.019) and targetoid HBP appearance (AUC: 0.68, 95% CI:0.57–0.79, *p* = 0.028). In the RFS analysis, patients with infiltrative appearance and targetoid HBP appearance showed significantly lower RFS rates than those without infiltrative appearance (2-year RFS rate, 48.0% vs. 72.0%; *p* = 0.009) and targetoid HBP appearance (2-year RFS rate, 60.0% vs. 35.0%; *p* = 0.003).

**Conclusion:**

An EOB-MRI model based on infiltrative appearance and targetoid HBP appearance showed good performance in predicting early recurrence of HCC after surgery, which may provide personalized guidance for clinical treatment decisions in patients with MVI-negative HCC.

## Key points

- Infiltrative appearance and targetoid HBP appearance were associated with early recurrence in patients with MVI-negative HCC.
- Based on the nomogram incorporating these risk factors, the MVI-negative HCC patients could be stratified into two subgroups with significantly different prognoses.
- The diagnostic model could identify a high risk of early recurrence in MVI-negative HCC and assist in prognosis evaluation.

Hepatocellular carcinoma (HCC) is the most common primary malignant tumor of the liver, and ranks as the third leading cause of cancer-related deaths in the world [1]. Although liver resection is the primary and effective treatment option for HCC, postoperative recurrence of HCC remains considerably high in the management of this disease, with a 5-year recurrence rate of approximately 50–70% after surgery [2, 3]. Notably, early recurrence within 2 years after surgery has been confirmed as an independent risk factor for treatment failure and poor prognosis, which may be related to alpha-fetoprotein (AFP), tumor size, and microvascular invasion (MVI) [3, 4].

Among them, clinical markers such as AFP and tumor size, while widely used, have limitations in predicting early recurrence. AFP levels can be elevated in non-malignant liver conditions (e.g., hepatitis B reactivation) and are not universally elevated in HCC [4]. Tumor size, though correlated with recurrence risk, does not capture the biological heterogeneity of tumors. In contrast, MVI is a well-established predictor, as it indicates the aggressive biological behavior of the tumor. The incidence of MVI in HCC ranges from 30 to 50%. However, approximately 50–70% of HCC patients have MVI-negative tumors, which complicates the identification of high-risk individuals [5].Numerous studies have demonstrated that postoperative recurrence is significantly higher and occurs more rapidly in MVI-positive HCC compared to MVI-negative cases [6–8]. However, studies regarding the prognostic determinants in MVI-negative HCC after surgery remain limited [9–12]. Notably, in clinical practice, there were still a subset of MVI-negative HCC patients present with early postoperative recurrence and poor prognosis, thus requiring urgently further exploration. The ability to predict early recurrence of MVI-negative HCC patients is crucial for postoperative management, as it helps identify high-risk patients who might benefit from closer surveillance or adjuvant therapies.

To date, there is emerging evidence that magnetic resonance imaging (MRI)—especially Gadoxetic acid-enhanced MRI (EOB-MRI)—may play an important role in the prognosis prediction of patients with HCC treated with surgical resection or interventional therapy, with its ability to assess tumor behavior, vascularity, and hepatic parenchymal changes [13, 14]. The Liver Imaging Reporting and Data System version 2018 (LI-RADS v2018) was a diagnostic algorithm established to standardize the imaging diagnosis and characterization of HCC in high-risk patients, which ensures reproducibility and reduces interobserver variability in imaging interpretation [15]. Recent studies have suggested that the LI-RADS features and categories may reflect tumor microenvironmental characteristics associated with aggressive behavior and postoperative prognosis in patients with HCC and other primary liver carcinomas [16, 17]. Existing models predominantly rely on postoperative pathological variables or different imaging markers, failing to address the biological heterogeneity of MVI-negative HCC or provide actionable preoperative insights [9–12]. Qu et al. [10] developed a model based on imaging features and histopathological grades showed a higher sensitivity in predicting early recurrence of MVI-negative HCC. Various qualitative and quantitative imaging features including arterial phase hyperenhancement (APHE), washout, mosaic architecture, peritumoral hypointensity on hepatobiliary phase (HBP), HBP hypointensity without arterial phase hyperenhancement, mild-moderate T2 hyperintensity, relative intensity ratio in HBP, lower relative enhancement ratio, can indicate early recurrence, but none are specific on their own [9, 11, 12]. This study aims to bridge this gap by leveraging LI-RADS v2018, a standardized imaging framework, to identify novel preoperative predictors of early recurrence in MVI-negative HCC.

Therefore, the aim of this study was to explore the role of LI-RADS v2018 features in predicting early recurrence (< 2 years) of MVI-negative solitary HCC after surgical resection.

## Materials and methods

This retrospective study was approved by the Institutional Review Board, and the requirement for patient written informed consent was waived.

### Patients

**Fig. 1:**
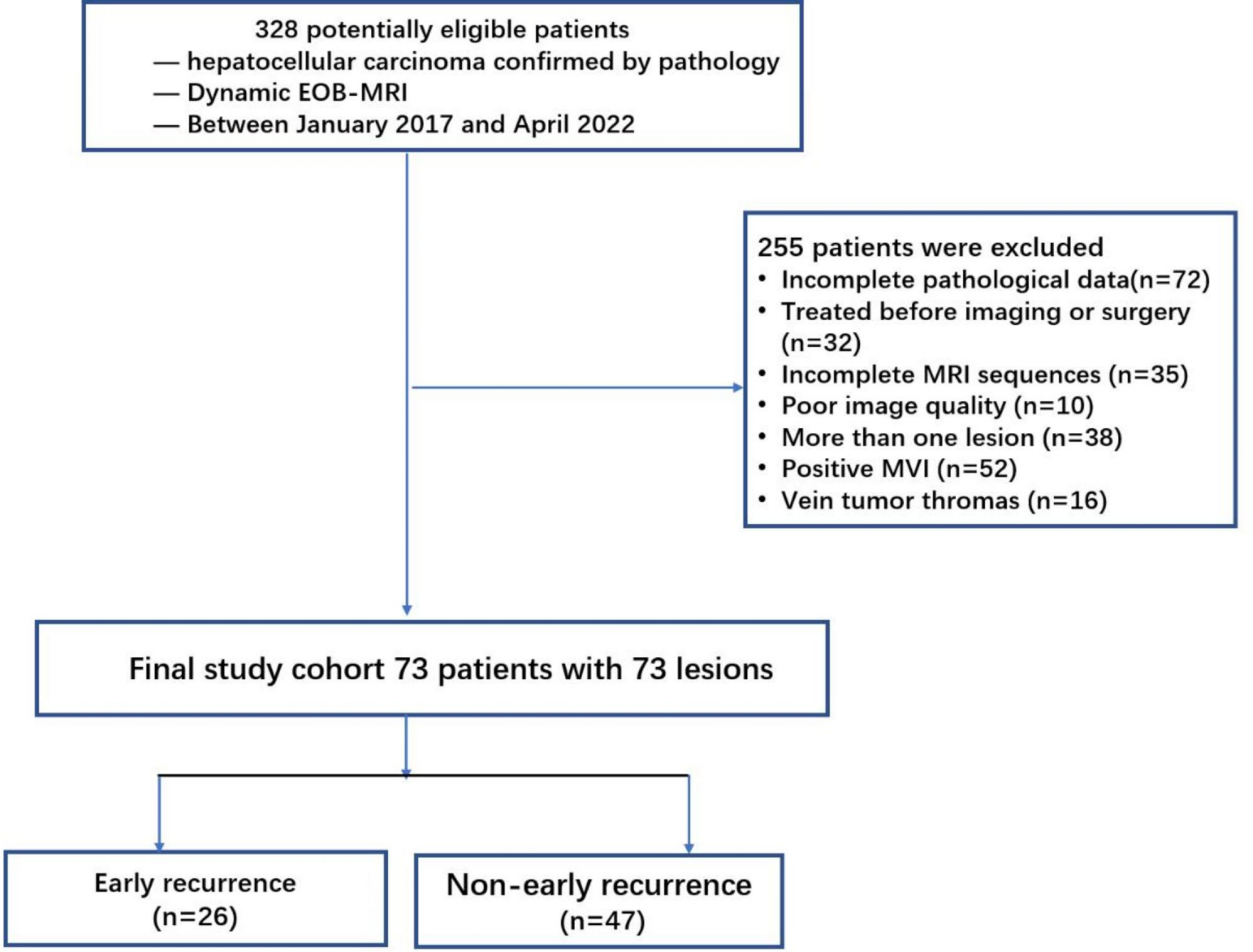
The patients’ recruitment pathway

From January 2017 to April 2022, we retrospectively identified 328 patients with a confirmed HCC who underwent curative hepatic resections in our institution. Inclusion criteria were as follows: (a) proven HCC via histopathologic assessment, and (b) patients who underwent EOB-MRI performed within 1 month before surgery. Patients were excluded according to the following: (a) incomplete pathological data, (b) patients treated before imaging or surgery, (c) MR images with incomplete sequences or poor image quality due to motion artifacts or poor resolution, (d) more than one HCC lesion, (e) postoperative histopathology confirming the existence of MVI, and (f) HCC with macrovascular invasion. Ultimately, 73 patients with MVI-negative HCC were enrolled in this study. The flow chart is shown in detail in Fig. 1.

### Imaging acquisition

MRI examinations were performed using two 3.0 T MRI scanners (Verio [Siemens Healthcare, Erlangen, Germany]; Ingenia [Philips Healthcare, Best, the Netherlands]). The MRI sequences included conventional in- and opposed-phase sequences, T1-weighted, T2-weighted, and diffusion-weighted sequences with b-values of 0, and 800 s/mm^2^. Dynamic T1-weighted imaging was obtained before and after intravenous administration of Gadoxetic acid (Primovist, Bayer Healthcare). Primovist administration was performed automatically using a power injector, through the cubital vein at a rate of 1–2 mL/s, for total dose of 0.025 mmoL/kg of body weight, followed by a 20 mL saline chaser. Detailed description for MRI protocol is provided in Table 1.

**Table 1:** Parameters of magnetic resonance imaging

	Sequences	TR(ms)	TE(ms)	Thickness(mm)	Matrix	FOV(mm)
Siemens Healthcare	T1WI	5.5	2.5	4	320 × 168	380 × 430
	T2WI	2000	74	5	320 × 161	380 × 430
	DWI	2200	73	5	128 × 78	380 × 430
	DCE	3.92	1.4	3	256 × 192	380 × 430
	HBP	3.92	1.4	3	320 × 182	380 × 430
Philips Healthcare	T1WI	140	1.2	4	280 × 189	450 × 300
	T2WI	2200	70	5	280 × 280	430 × 380
	DWI	2226	84	5	152 × 122	430 × 380
	DCE	4.2	1.5	4	180 × 200	400 × 300
	HBP	4.2	1.5	4	320 × 200	400 × 300

Abbreviations TR, repetition time; TE, echo time; DCE, dynamic contrast-enhanced; HBP, hepatobiliary phase

### Imaging analysis

MR images were reviewed based on LI-RADS v2018 independently by two abdominal radiologists (Liang* and Han*, with 11 and 8 years of MRI experience, respectively). These radiologists were aware of the presence of HCC, but were blinded to the clinical, laboratory, and follow-up results. In cases of disagreement between the two radiologists, a joint review session was conducted. During this session, both radiologists re-evaluated the imaging features in question, discussed the LI-RADS v2018 criteria, and resolved discrepancies by reaching a consensus. This consensus reading was then used for further analysis in order to ensure consistency and minimize variability in the interpretation of imaging features. The features of LI-RADS 2018 were categorized into major features, ancillary features (particularly prone to HCC), ancillary features (particularly prone to malignancy, not HCC), and LR-M features, in order to evaluate each lesion. Threshold or sub-threshold growth, and US visibility as a discrete nodule were excluded from the study. Other imaging features including intratumoral artery, satellite nodules, and peritumoral enhancement were also evaluated.

### Clinical evaluation

The following clinical characteristics were collected from the patients’ electronic medical records: gender, age, Edmondson grade, AFP, hepatitis B virus (HBV), Child-Pugh grade, albumin-bilirubin index (ALBI), alanine aminotransferase, serum total bilirubin, plasma albumin, prothrombin time, and platelets.

### Follow-up surveillance

Abdominal ultrasound, contrast-enhanced computed tomography (CT), or MRI was performed every 3–6 months after surgery to investigate the tumor recurrence. Early recurrence was defined as the appearance of new tumor nodules within 2 years, which exhibited typical imaging features of HCC, and were diagnosed by at least two imaging modalities or pathologic results. Follow-up was evaluated until April 2024 or until recurrence occurred.

### Statistical analysis

The baseline characteristics were compared using a Student’s t-test (two-sided) or Mann–Whitney U test for continuous variables, and chi-square tests or Fisher’s exact tests for categorical variables. The inter-reader agreements between the MRI features were analyzed using Cohen’s *k* statistics as follows: poor agreement, *k =* 0.00–0.20; fair, *k =* 0.21–0.40; moderate, *k =* 0.41–0.60; good, *k =* 0.61–0.80; and excellent, *k =* 0.81–1.00. The significant variables identified via the univariate Cox analysis, with a significance level of p-value < 0.1, were further selected for a multivariate Cox regression analysis. A nomogram was subsequently constructed based on the predictors, with a p-value < 0.05 in the multivariate analysis. The diagnostic performance was evaluated using receiver operating characteristic (ROC) analyses. The Kaplan–Meier method was used to estimate the recurrence-free survival (RFS) rates. Statistical analyses were performed using SPSS version 20.0 (IBM, Armonk, NY) and R software (version 3.5.3). A p-value of less than 0.05 was considered statistically significant.

## Results

### Patient and tumor characteristics

Based on the selection criteria, a total of 73 patients with 73 MVI-negative HCCs were included. Of the patients included in this study, 26 experienced early recurrence (35.6%) and 47 relapsed at the end of follow-up (64.4%). The characteristics of the patients and tumors are shown in Table 2. No significant differences were found between the early recurrence group and no early recurrence group in terms of any clinical factors.

**Table 2:** Clinical and pathological characteristics of early recurrence and non-early recurrence groups

Variable	Non-ER(*n* = 47)	ER(*n* = 26)	*P*
**Gender (%)**			0.585
Male	37(78.7%)	19(73.1%)	
Female	10(21.3%)	7(26.9%)	
**Age (IR**,** ranges) (year)**	62(49,71)	58(49,66)	0.384
**HBV infection (%)**			0.111
Negative	13(27.7%)	3(11.5%)	
Positive	34(72.3%)	23(88.5%)	
**Child-Pugh (%)**			0.454
A	46(97.9%)	26(100.0%)	
B	1(2.1%)	0(0.0%)	
**AFP (≥ 400ng/ml)**			0.143
Negative	30(63.8%)	12(46.2%)	
Positive	17(36.2%)	14(53.8%)	
**Maximum diameter**			0.162
**<5 cm**	33(70.2%)	14(53.8%)	
**≥ 5 cm**	14(29.8%)	12(46.2%)	
**ALT (IR**,** U/L)**	30(22,67)	54(30,98)	0.051
**STB (IR**,** umol/L)**	17.1(14.8,23.9)	18.5 (15.9,31.3)	0.457
**PA (M ± SD**,** g/L)**	38.44 ± 5.12	37.82 ± 6.86	0.663
**PT (IR**,** s)**	13.9 (13.1,14.6)	13.7 (13.2,14.4)	0.489
**Platelet (IR**,** ×10**^**9**^**/L)**	174 (137,260)	212 (155,245)	0.531
**ALBI**			0.739
1	9 (19.1%)	5 (19.2%)	
2	35 (74.5%)	18 (69.2%)	
3	3 (6.4%)	3 (11.6%)	
**Edmonson-Steiner grade**			0.228
** I-II**	32(68.1%)	14(53.8%)	
** III-IV**	15(31.9%)	12(46.2%)	

Abbreviations AFP, alpha-fetoprotein; ALBI, albumin-bilirubin Index; ALT, alanine aminotransferase; ER, early recurrence; HBV, Hepatitis B; IR, interquartile range; M, mean; PA, plasma albumin; PT, prothrombin time; STB, serum total bilirubin; SD, standard deviation

### Univariate analysis of independent predictors associated with early recurrence

The inter-observer agreement of MRI features is shown in Table 3. It was good to excellent (*κ* = 0.652–0.945) for LI-RADS features and other MR imaging features.

**Table 3:** Univariate analysis of MRI LI-RADS and other imaging features for predicting early recurrence of solitary MVI negative HCC

Characteristic	Univariate analysis		*P* value	Kappa(95%CI)	
	Non-ER (*n* = 47)	ER(*n* = 26)			
**Major features of HCC**					
Non-rim APHE			0.185	0.908 (0.806, 1.000)	
Negative	18(38.3%)	6(23.1%)			
Positive	29(61.7%)	20(76.9%)			
Nonperipheral washout			0.134	0.855 (0.696, 1.000)	
Negative	10(21.3%)	2(7.7%)			
Positive	37(78.7%)	24(92.3%)			
Enhancing capsule			0.424	0.870 (0.747, 0.993)	
Negative	15(31.9%)	6(23.1%)			
Positive	32(68.1%)	20(76.9%)			
**Ancillary features that favor HCC over non-HCC malignancies**					
Nonenhancing capsule			0.368	0.819 (0.622, 1.000)	
Negative	43(91.5%)	22(84.6%)			
Positive	4(8.5%)	4(15.4%)			
Nodule-in-nodule			0.667	0.793 (0.399, 1.000)	
Negative	46(97.9%)	25(96.2%)			
Positive	1(2.1%)	1(3.8%)			
Mosaic architecture			**0.057**	0.834 (0.707, 0.960)	
Negative	29(61.7%)	10(38.5%)			
Positive	18(38.3%)	16(61.5%)			
Blood products in mass			0.142	0.887 (0.763,1.000)	
Negative	38(80.9%)	17(65.4%)			
Positive	9(19.1%)	9(34.6%)			
Fat in mass, more than adjacent liver			0.381	0.906 (0.779,1.000)	
Negative	40(85.1%)	20(76.9%)			
Positive	7(14.9%)	6(23.1%)			
**Ancillary features favoring malignancies in general**,** not HCC in particular**					
Mild-moderate T2 hyperintensity			0.126	0.882 (0.653, 1.000)	
Negative	4(8.5%)	0(0.0%)			
Positive	43(91.5%)	26(100.0%)			
Restricted diffusion			0.176	0.660 (0.039,1.000)	
Negative	0(0.0%)	1(3.8%)			
Positive	47(100.0%)	25(96.2%)			
Corona enhancement			0.251	0.652(0.203,1.000)	
Negative	46(97.9%)	24(92.3%)			
Positive	1(2.1%)	2(7.7%)			
Fat sparing in solid mass			0.188	0.850(0.562,1.000)	
Negative	44(93.6%)	26(100.0%)			
Positive	3(6.4%)	0(0.0%)			
Iron sparing in solid mass			0.286	0.793(0.399,1.000)	
Negative	45(95.7%)	26(100.0%)			
Positive	2(4.3%)	0(0.0%)			
Transitional phase hypointensity			0.682	0.801(0.585,1.000)	
Negative	5(10.6%)	2(7.7%)			
Positive	42(89.4%)	24(92.3%)			
Hepatobiliary phase hypointensity			0.126	0.902 (0.711,1.000)	
Negative	4(8.5%)	0(0.0%)			
Positive	43(91.5%)	26(100.0%)			
**LR-M features**					
Rim APHE			**0.072**	0.823(0.657, 0.990)	
Negative	42(89.4%)	19(73.1%)			
Positive	5(10.6%)	7(26.9%)			
Peripheral “washout”			0.238	0.819(0.575,1.000)	
Negative	45(95.7%)	23(88.5%)			
Positive	2(4.3%)	3(11.5%)			
Delayed central enhancement			0.238	0.748(0.478,1.000)	
Negative	45(95.7%)	23(88.5%)			
Positive	2(4.3%)	3(11.5%)			
Targetoid restriction			0.442	0.842(0.630,1.000)	
Negative	44(93.6%)	23(88.5%)			
Positive	3(6.4%)	3(11.5%)			
Targetoid TP			**0.061**	0.871(0.928,1.000)	
Negative	41(87.2%)	18(69.2%)			
Positive	6(12.8%)	8(30.8%)			
Targetoid HBP			**0.001**	0.937(0.850,1.000)	
Negative	39(83.0%)	12(46.2%)			
Positive	8(17.0%)	14(53.8%)			
Infiltrative appearance			**0.004**	0.918 (0.827, 1.000)	
Negative	29(61.7%)	7(26.9%)			
Positive	18(38.3%)	19(73.1%)			
Marked diffusion restriction			0.123	0.806(0.661, 0.952)	
Negative	15(31.9%)	4(15.4%)			
Positive	32(68.1%)	22(84.6%)			
Necrosis or severe ischemia			0.587	0.945 (0.870, 1.000)	
Negative	23(48.9%)	11(42.3%)			
Positive	24(51.1%)	15(57.7%)			
**Other MRI imaging features**					
Intratumoral artery			0.115	0.892 (0.772,1.000)	
Negative	37(78.7%)	16(61.5%)			
Positive	10(21.3%)	10(38.5%)			
Satellite nodules			0.304	0.793 (0.636, 0.951)	
Negative	36(76.6%)	17(65.4%)			
Positive	11(23.4%)	9(34.6%)			
Peritumoral enhancement			0.368	0.777 (0.535,1.000)	
Negative	43(91.5%)	22(84.6%)			
Positive	4(8.5%)	4(15.4%)			

Abbreviations APHE, arterial phase hyperenhancement; CI: confidence interval; HBP: hepatobiliary phase; OR, odds ratio; TP: transitional phase

The imaging features of HCC are summarized in Table 3. In the univariate regression analysis, relevant predictive factors were selected based on a significance level of *p* < 0.1. Five factors demonstrated a significant correlation with the early recurrence of HCC, including: mosaic architecture (*p* = 0.057), infiltrative appearance (*p* = 0.004), rim arterial phase hyperenhancement (APHE, *p* = 0.072), targetoid transitional phase appearance (TP, *p* = 0.061), and targetoid hepatobiliary phase appearance (HBP, *p* = 0.001).

### Multivariate analysis of independent predictors associated with early recurrence

**Fig. 2:**
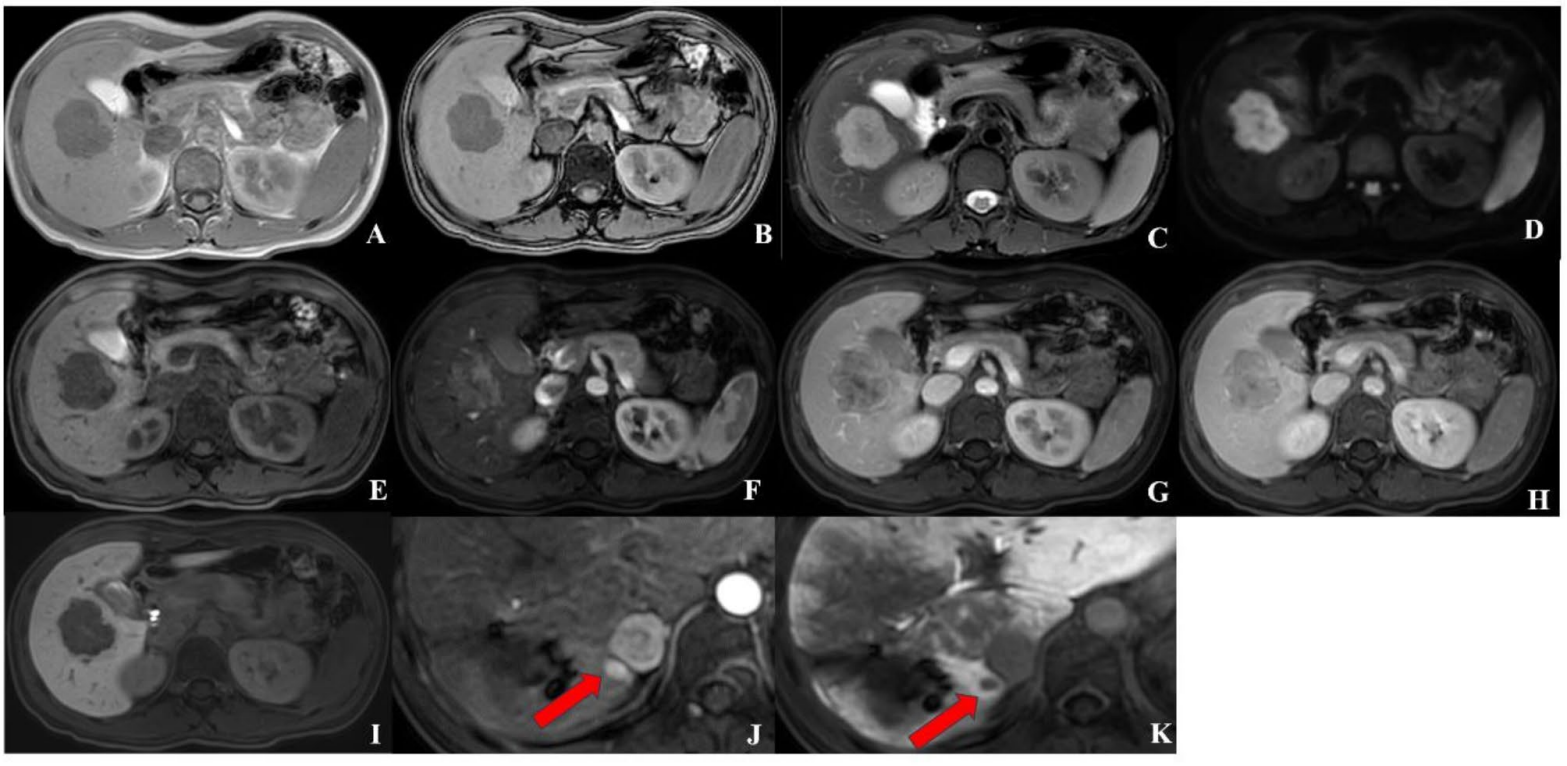
26-year-old woman with surgically proven HCC in segment V. (**A**-**B**) T1-weighted images in phase and out phase showing hypointensity; (**C**) T2-weighted images showing hyperintensity; (**D**) DWI showing hyperintensity; (**E**)Pre-contrast T1-weighted images showing hypointensity; (**F**) Arterial phase images showing heterogeneous arterial hyperenhancement (APHE) with infiltrative appearance; (**G**) Portal venous phase showing nonperipheral washout; (**H**) Transitional phase showing hypointensity; (**I**) Hepatobiliary phase showing targetoid appearance of peripheral low signal and high signal in the center; (**J**-**K**) A new nodule with diameter of 8 mm in segment VI-VII after surgery showing APHE on arterial phase and low signal on hepatobiliary phase (Red arrow)

In the multivariate analysis, infiltrative appearance (hazard ratio [HR]: 4.237; *p* = 0.023) and targetoid HBP appearance (HR: 14.958; *p* = 0.018) were demonstrated to have independent predictive impacts on early recurrence (Table 4) (Fig. 2).

**Table 4:** Multivariate analysis of MRI LI-RADS and other imaging features for predicting early recurrence of solitary MVI negative HCC

Imaging features	B	*P*	OR	95%CI	
Mosaic architecture	0.932	0.110	2.539	0.811	7.957
Infiltrative appearance	1.444	0.023	4.237	1.224	14.67
Rim APHE	-0.929	0.367	0.395	0.053	2.966
Targetoid TP	-1.059	0.347	0.347	0.038	3.156
Targetoid HBP	2.705	0.018	14.958	1.596	140.204

Abbreviations B, regression coefficients; APHE, arterial phase hyperenhancement; CI: confidence interval; HBP: hepatobiliary phase; OR, odds ratio; TP: transitional phase

### Diagnostic model development

**Fig. 3:**
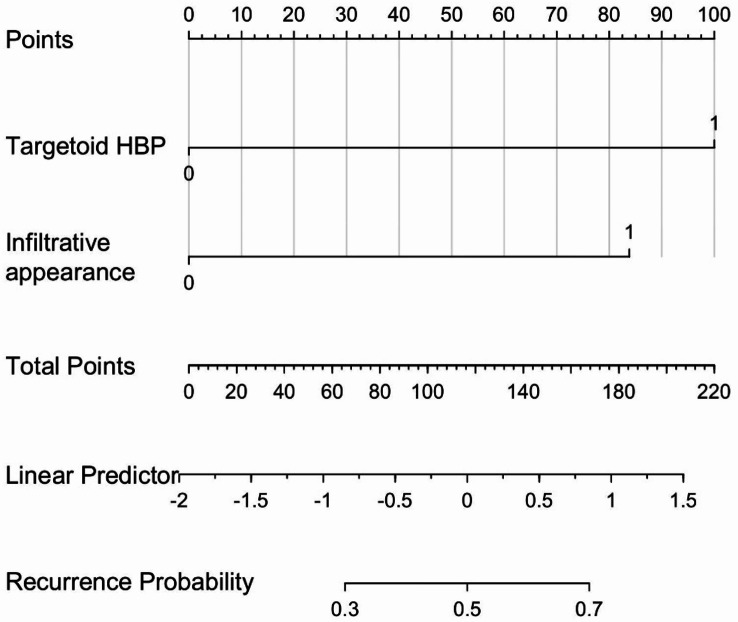
Nomogram to predict the probability of early recurrence of patients with solitary MVI negative HCC after surgery

**Fig. 4:**
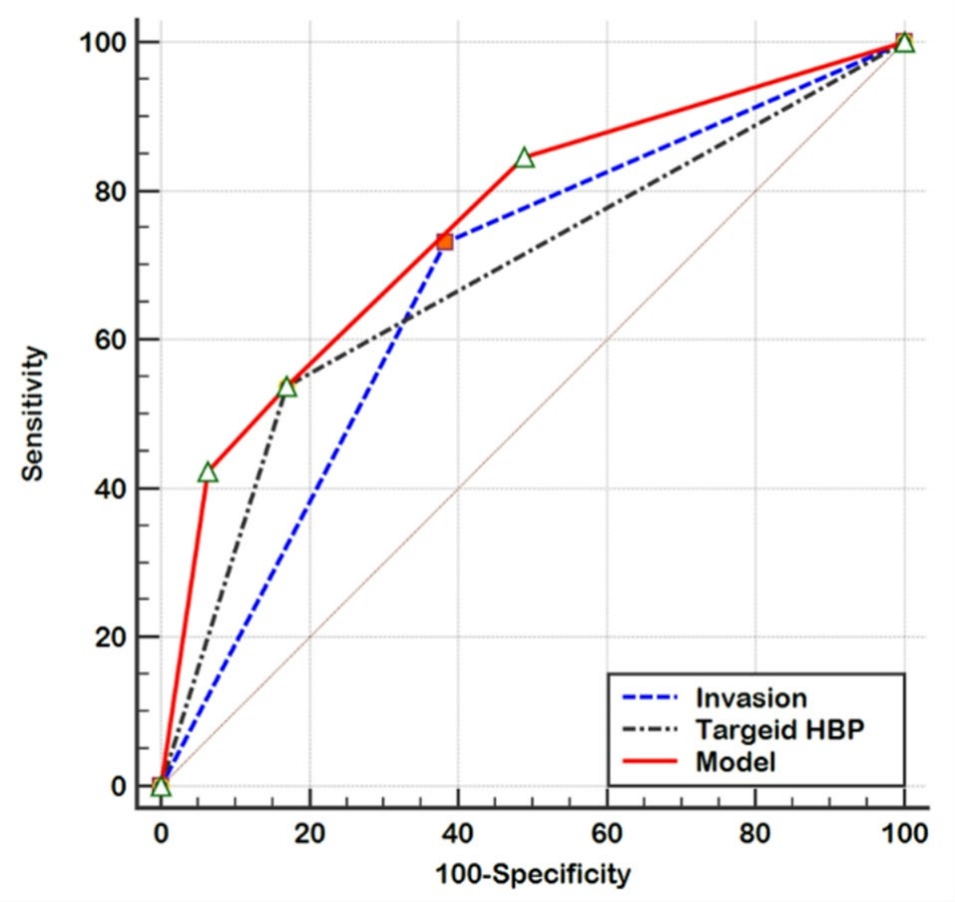
The ROC curves of the model

A diagnostic model was established using the independent predictors of early recurrence, including infiltrative appearance and targetoid HBP appearance, and the diagnostic performance of the model was analyzed (Table 5). The nomogram for the model was shown in Fig. 3. The AUC value of the model combining these two features was 0.76 (95% CI: 0.64–0.85), which demonstrated a significant improvement in prediction compared to infiltrative appearance alone (0.67, 95% CI: 0.55–0.78, *p* = 0.019) or targetoid HBP appearance alone (0.68, 95% CI:0.57–0.79, *p* = 0.028) (Fig. 4). The model demonstrated significantly higher predictive accuracy for larger tumors (> 5 cm) compared to smaller tumors (≤ 5 cm), with AUC values of 0.845 (95% CI: 0.72–0.94) versus 0.684 (95% CI: 0.55–0.81), respectively (*p* = 0.069).

**Table 5:** Predictive performance of the model

Appearance	AUC	95%CI	Sensitivity	95%CI	Specificity	95%CI
Infiltrative appearance	0.67	0.55, 0.78	73.08	52.20, 88.40	61.70	46.40, 75.50
Targetoid HBP	0.68	0.57, 0.79	53.85	33.40, 73.40	82.98	69.20, 92.40
Model	0.76	0.64, 0.85	53.85	33.40, 73.40	82.98	69.20, 92.40

Abbreviations AUC: the areas under the receiver operating characteristic curves; CI: confidence interval; HBP: hepatobiliary phase; Model: infiltrative appearance and targetoid HBP

### RFS rates based on the infiltrative appearance and targetoid HBP appearance

**Fig. 5:**
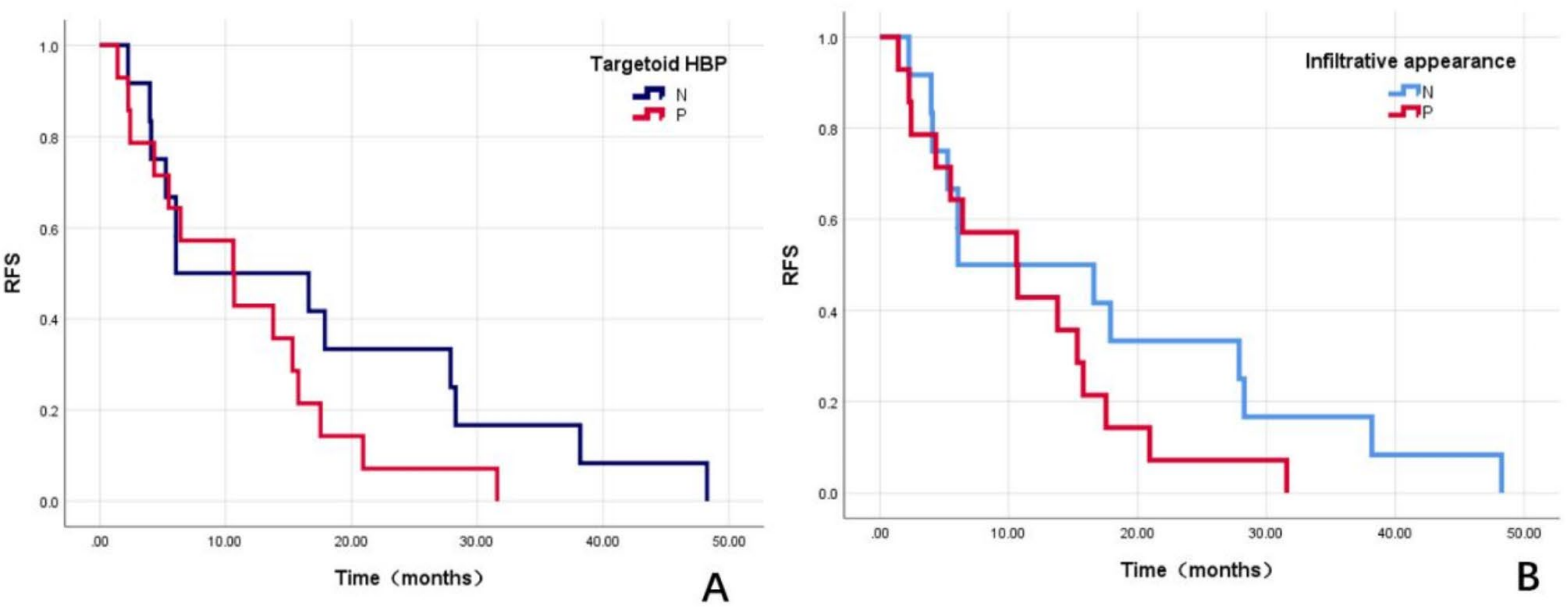
Recurrence-free survival (RFS) rates of patients with solitary MVI negative HCC after surgery based on targetoid HBP (**A**) and infiltrative appearance (**B**)

The mean follow-up period was 14.0 months (range, 1.4–48.3 months). RFS rates based on the infiltrative appearance and targetoid HBP appearance are shown in Fig. 5. Patients with infiltrative appearance and targetoid HBP appearance showed significantly lower RFS rates than those without infiltrative appearance (2-year RFS rate, 48.0% vs. 72.0%; *p* = 0.009) and targetoid HBP appearance (2-year RFS rate, 60.0% vs. 35.0%; *p* = 0.003).

## Discussion

In this study, we constructed a diagnostic model for predicting the early recurrence of MVI-negative HCC using MRI LI-RADS v2018 features. The results indicated that infiltrative appearance and targetoid HBP appearance were significant risk factors for early recurrence of MVI-negative HCC. The model showed satisfactory prediction results with an AUC of 0.76 (95% CI: 0.64–0.85), which was significantly higher than that of infiltrative appearance (AUC: 0.67, 95% CI: 0.55–0.78) and targetoid HBP appearance (AUC: 0.68, 95% CI: 0.57–0.79). It can be used as a simple and easy method of prognostic prediction in routine clinical practice.

In this study, infiltrative appearance—indicative of malignancy but not HCC-specific, according to LI-RADS v2018—was an independent risk factor of early recurrence in HCC, which is consistent with the results of previous studies [13, 18]. The infiltrative appearance is an aggressive imaging feature, described as infiltration of tumor cells into the liver parenchyma. This suggests a permeative pathological growth pattern, which has been associated with more aggressive tumor behavior, and a worse prognosis of HCC [13, 18]. It can also help identify some aggressive phenotypes of HCC, such as CK19-positive HCC. Hence, this imaging feature is highly suggestive of proliferative HCC.

According to the LI-RADS categories, targetoid HBP was not a major feature of HCC. Rather, it was an imaging feature of cholangiocarcinoma or combined hepatocellular cholangiocarcinoma, which refers to a target-like morphology that reflects peripheral hypercellularity and central fibrosis, or ischemia within the tumor. In our study, 30.1% of patients in MVI-negative HCC showed targetoid HBP, which is significantly higher than the 4.9% reported by Park et al., and lower than the 74.5-77.1% reported by Wang, with no mention of the MVI status [19]. A previous study has reported that HCC with targetoid HBP expresses a higher Ki-67 index, which is associated with tumor invasiveness, and poorer prognoses in patients with HCC [20]. Although evidence supporting the prognostic value of targetoid HBP is under-recognized and scarce, MVI-negative HCC with targetoid HBP seems to be more invasive, and the value of targetoid HBP needs to be further explored.

A study has found that tumor size, and mosaic architecture are related to a worse prognosis in MVI-negative HCCs [9]. Another study has also revealed that tumor size, APHE, washout, and mosaic architecture are risk predictors of recurrence-free survival in solitary MVI-negative HCCs ≤ 5 cm [11]. In our study, the known imaging features are not associated with postsurgical recurrence, which is contrary to the previous reports. Tumor size is one of the most easily assessable prognostic factors for HCC, and has been correlated with MVI, differentiation, and postsurgical recurrence [8, 21, 22]. The discrepancy in tumor size may be attributed to the different guidelines used, with varying cutoff values used from continuous variables to categorical variables. Additionally, a study [23] has demonstrated that mosaic architecture is an independent factor for MVI but not recurrence, which was consistent with our study. As a major imaging feature, several studies have found that APHE and washout—representing the vascular composition of the tumor—is also an independent predictor of MVI, tumor differentiation, and early recurrence of HCC, which was inconsistent with our study [11, 24–27].

Although infiltrative appearance and targetoid HBP appearance were significant predictors of early recurrence in MVI-negative patients, their diagnostic performances were only fair, with an AUC of less than 70%. Model-diagnosed early recurrence of MVI-negative HCCs showed a higher AUC than the infiltrative appearance and targetoid HBP appearance. Wei et al. [13] developed a preoperative MRI-based recurrence risk score that incorporated imaging features such as tumor size, APHE, and washout, achieving an AUC of 0.73 for predicting early recurrence. Similarly, Chen et al. [14] combined preoperative MRI features with clinical parameters to predict early recurrence of HCC, reporting an AUC of 0.78. While these studies demonstrated the utility of imaging features in prognostic models, our study uniquely focuses on MVI-negative HCC and highlights the predictive value of infiltrative appearance and targetoid HBP appearance, which are not commonly included in existing models. Our model achieved an AUC of 0.76, which is comparable to these combined imaging-clinical models, suggesting that LI-RADS v2018 features alone can provide robust prognostic information without the need for additional clinical parameters. To our knowledge, only one study constructed a prognostic nomogram using LI-RADS imaging and clinical features for predicting the prognosis of patients with solitary MVI-negative HCC ≤ 5 cm, with C-index values of 0.713 and 0.707 in the development and validation cohorts, respectively [11]. Therefore, the model in our study could address this gap in predicting high-risk early recurrence of MVI-negative patients with individualized patient management such as follow-ups, and post-operative adjuvant treatment using TACE or molecular targeted agents.

For postoperative survival stratification, infiltrative appearance and targetoid HBP appearance were found significantly correlated with poorer postoperative prognosis in the current study, which was in line with previous studies [28, 29]. Our diagnostic model, which incorporates infiltrative appearance and targetoid HBP appearance, has several potential clinical applications in the management of MVI-negative HCC. First, the model could be used to stratify patients into high-risk and low-risk subgroups for early recurrence, enabling more personalized postoperative surveillance strategies. More frequent imaging follow-up (e.g., every 3 months) will be recommended for the high-risk patients, while low-risk patients could follow standard surveillance protocols. Second, the model could be integrated into decision-support systems to assist clinicians in making evidence-based treatment decisions. By incorporating LI-RADS v2018 imaging features into existing HCC management algorithms, our model could enhance the precision of prognostic assessments and improve patient outcomes of MVI-negative patients.

This study has several limitations. First, this was a retrospective and single-center study with limited sample size which may restrict generalizability. While the results are promising, the findings need to be validated in larger, prospective and multi-center cohorts to assess the external applicability of the model. Additionally, the study lacks an external validation cohort, which is essential to confirm the reproducibility of the model in diverse patient populations and across varying imaging settings. Without this external validation, it remains uncertain whether the model’s performance will hold up when applied to different clinical settings or healthcare systems. Further research is required to validate the model in independent cohorts before it can be recommended for widespread use in clinical practice.

Second, in our study, the model presented here focuses only on imaging features because there was no significant clinical, labarotary and histological factors such as AFP levels, Child-Pugh grade, Edmondson grade and so on, which may provide further insights into tumor biology and improve prediction accuracy. Therefore, more key clinical markers such as AFP levels, genetic mutations, or immune markers should be incorporated with imaging features in order to enhance the model’s prognostic power and create a more comprehensive, multi-modal prediction tool for early recurrence of MVI-negative HCC in the future.

Third, the exclusion of patients with macrovascular invasion, multiple tumors, incomplete imaging data and nonsurgical treatment in our study may introduce selection bias, therefore precluding the generalizability of the results in broader clinical scenarios. The model’s predictive accuracy and utility in these excluded populations remain uncertain, and further studies should include a wider range of patients to assess the model’s robustness and generalizability in these subgroups.

Fourth, early recurrence time was defined in different studies as recurrence occurring within one or two years [14, 30, 31]. In the future, it would be beneficial to evaluate the imaging risk factors in predicting the later recurrence of HCCs in the longer follow-up.

Finally, the current study does not explore more advanced prediction methods such as machine learning or combined radiomic-clinical models, which have the potential to improve prediction accuracy by incorporating a larger number of variables and capturing complex, non-linear relationships between tumor characteristics and clinical outcomes. Therefore, future research should explore these methods to determine if they can further enhance the predictive power of early recurrence in MVI-negative HCC, potentially offering more personalized and effective clinical decision-making tools.

In conclusion, this study highlights the value of an EOB-MRI nomogram incorporating LI-RADS v2018 features, specifically infiltrative appearance and targetoid HBP appearance, in predicting early recurrence of MVI-negative HCC patients after surgical resection. By providing a more accurate, non-invasive method for predicting early recurrence, the model offers a powerful tool for clinicians to identify high-risk patients, tailor postoperative surveillance intervals and personalize treatment strategies, potentially leading to better outcomes in MVI-negative patients.

## Data Availability

No datasets were generated or analysed during the current study.

## Abbreviations

AFP: Alpha-fetoprotein
AP: Arterial phase
APHE: Arterial phase hyperenhancement
CT: Computed tomography
EOB-MRI: Gadoxetic acid–enhanced magnetic resonance imaging
HBV: Hepatitis B virus
HBP: Hepatobiliary phase
HCC: Hepatocellular carcinoma
LI-RADS v2018: Liver Imaging Reporting and Data System version 2018
MVI: Microvascular invasion
PVP: Portal venous phase
TP: Transitional phase
